# eHealth for patients with rare diseases: the eHealth Working Group of the European Reference Network on Rare Multisystemic Vascular Diseases (VASCERN)

**DOI:** 10.1186/s13023-020-01604-4

**Published:** 2021-04-08

**Authors:** Alessia Paglialonga, Raffaella Gaetano, Leema Robert, Marine Hurard, Luisa Maria Botella, Natasha Barr, Guillaume Jondeau, Alessandro Pini

**Affiliations:** 1 Institute of Electronics, Information Engineering and Telecommunications (IEIIT), Italian National Research Council (CNR), Milan, Italy; 2 Italian National Research Council (CNR), Institute of Biomedical Research and Innovation (IRIB), Palermo, Italy; 3 Department of Clinical Genetics, VASCERN HTAD European Reference Centre, Guys and St Thomas NHS Foundation Trust, London, UK; 4 VASCERN Coordination Project-Team, Assistance Publique-Hôpitaux de Paris Hôpital Bichat-Claude Bernard, VASCERN HTAD European Reference Centre, Paris, France; 5 Asociación HHT España, Almansa, Spain; 6 VASCERN European Patient Advocacy Group (ePAG), Paris, France; 7 VASCERN Coordinator, Cardiology Department, Reference Center for Marfan Syndrome and Related Diseases, INSERM U1148 LVTS, Assistance Publique-Hôpitaux de Paris, Université de Paris, Hôpital Bichat-Claude Bernard, VASCERN HTAD European Reference Centre, Paris, France; 8 Cardiovascular-Genetic Center, IRCCS Policlinico San Donato, Via Morandi 30, 20097 San Donato Milanese, MI Italy; 9 VASCERN eHealth Working Group, Past Chair, Paris, France

**Keywords:** European reference network, European commission 3rd health programme, eHealth, Knowledge exchange, mHealth, Orphan diseases, Rare diseases, Telemedicine, Vascular diseases

## Abstract

**Background:**

The European Reference Network on Rare Multisystemic Vascular Diseases (VASCERN) was launched in 2017 and involves, to date, 35 highly specialised multidisciplinary expert centres (from the 30 full Healthcare Provider members) coming from 11 countries and more than 70 patient organizations from 16 countries. The eHealth Working Group (WG) of VASCERN was set up to develop practical, patient-centred solutions and strategies for effective use of eHealth tools to answer the needs of patients with multisystemic vascular rare diseases.

**The eHealth WG:**

Following the identified patients’ needs and following the guiding principles of collaboration and patient-centredness, the eHealth WG was created with the following aims: to develop a mobile app to help patients find expert centres and patient organizations, and to develop resources (Pills of Knowledge, PoK) for training and education via digital platforms (eLearning). The mobile app includes, to date, functionalities that allow users to find expert centres and patient organizations across Europe in the area of rare multisystemic vascular diseases. Discussed app developments include personalized digital patient passports, educational material, emergency management guidelines and remote consultations. Regarding training and education, a variety of PoK have been developed. The PoK cover several topics, target several user groups, and are delivered in various formats so that they are easy-to-use, easy-to-understand, informative, and viable for delivery and sharing through digital platforms (eLearning) including, e.g., the VASCERN YouTube™ channel.

**Conclusion:**

Overall, the work carried out by the eHealth WG of VASCERN can be seen as a pilot experience that may serve as a basis to for collaborative development of patient-centred eHealth tools that answer the needs of patients with various rare diseases, not limited to rare multisystemic vascular diseases. By expanding the multidisciplinary approach here described, clinical and research networks can take advantage of eHealth services and use them as strategic assets in achieving the ultimate goal of ensuring equity of access to prevention programs, timely and accurate diagnosis and specialized care for patients with rare diseases throughout Europe.

## Background

The ongoing digital health revolution, supported by ubiquitous connectivity, enables new ways of delivering decentralized healthcare services through eHealth solutions. The use of eHealth in the area of rare diseases (RD) holds promise to support service delivery and improve quality of care as well as patients’ self-efficacy and quality of life. Patients with RDs may experience difficulties in finding appropriate clinical expertise and they frequently have to travel long distances for their care, often facing language and cultural barriers as well as substantial financial burden [1, 2]. Because of the intrinsic characteristics of RDs (e.g., large number of disorders and syndromes, low individual prevalence, severity, often limited information, and scarcity of therapies) the area of RD care can indeed benefit greatly from cross-border collaboration and from meaningful use of eHealth [3].

eHealth has the potential to provide innovative solutions to health issues and may be viewed as a key ‘enabling’ technology to improve care and the experience of care for those living with RDs. European policies promote the implementation and uptake of eHealth tools, supporting the development of digital infrastructures to facilitate cross-border health services and cooperation [4]. The European eHealth Action Plan 2012–2020 provides a roadmap to empower patients, HCPs, and citizens through modern devices and technologies, including a special focus on mobile health (mHealth) [5]. The eHealth Action Plan supports the use of eHealth to enable personalised, citizen centred healthcare, which can help reduce errors and costs, improve outcomes and quality of life, and decrease inequalities [5]. The eHealth Network is a voluntary network connecting national authorities responsible for eHealth set up by the EU to facilitate cooperation and information exchange across Europe [6], for example in the areas of cross-border exchange of health data including interoperability issues, privacy protection, standardisation, and mHealth [7]. The current ongoing discussion about eHealth and RDs is mainly focused on patient registries, telemedicine, and issues related to data sharing for clinical or research purposes [3, 8]. In addition to these strategical themes, it would also be important to develop new, patient-centered frameworks for the development of eHealth tools that can support patient awareness and knowledge.

In this article, based on our experience within the European Reference Network for Vascular Rare Diseases (VASCERN), we discuss how practical eHealth solutions can be implemented within a multidisciplinary European collaborative network for the benefit of patients with RDs. We present our approach to collaborative development of patient-centred eHealth tools that can support patient awareness and cross-border healthcare and that may be used as a reference model to build scalable eHealth frameworks in the area of RDs.

## VASCERN: The European Reference Network for Vascular Rare Diseases

To support sharing of expertise and cooperation in the area of RDs, the EU has launched the European Reference Networks (ERNs) [3, 8]. ERNs pool highly specialised healthcare providers (HCPs) across Europe with expertise on rare or low prevalence complex diseases or conditions. The aim of the ERNs is to promote the sharing of expertise and facilitate cross-border consultation of the patients in reference centres, whenever necessary, to assure equity in access to healthcare for patients with RDs throughout Europe [1, 6, 9–16]. The first 24 ERNs were launched in 2017 as virtual networks enabling HCPs across Europe to access and share expertise for the care of patients with complex or rare disorders, and they gathered at launch more than 300 hospitals and 900 highly specialised teams across Europe [17].

**Fig. 1:**
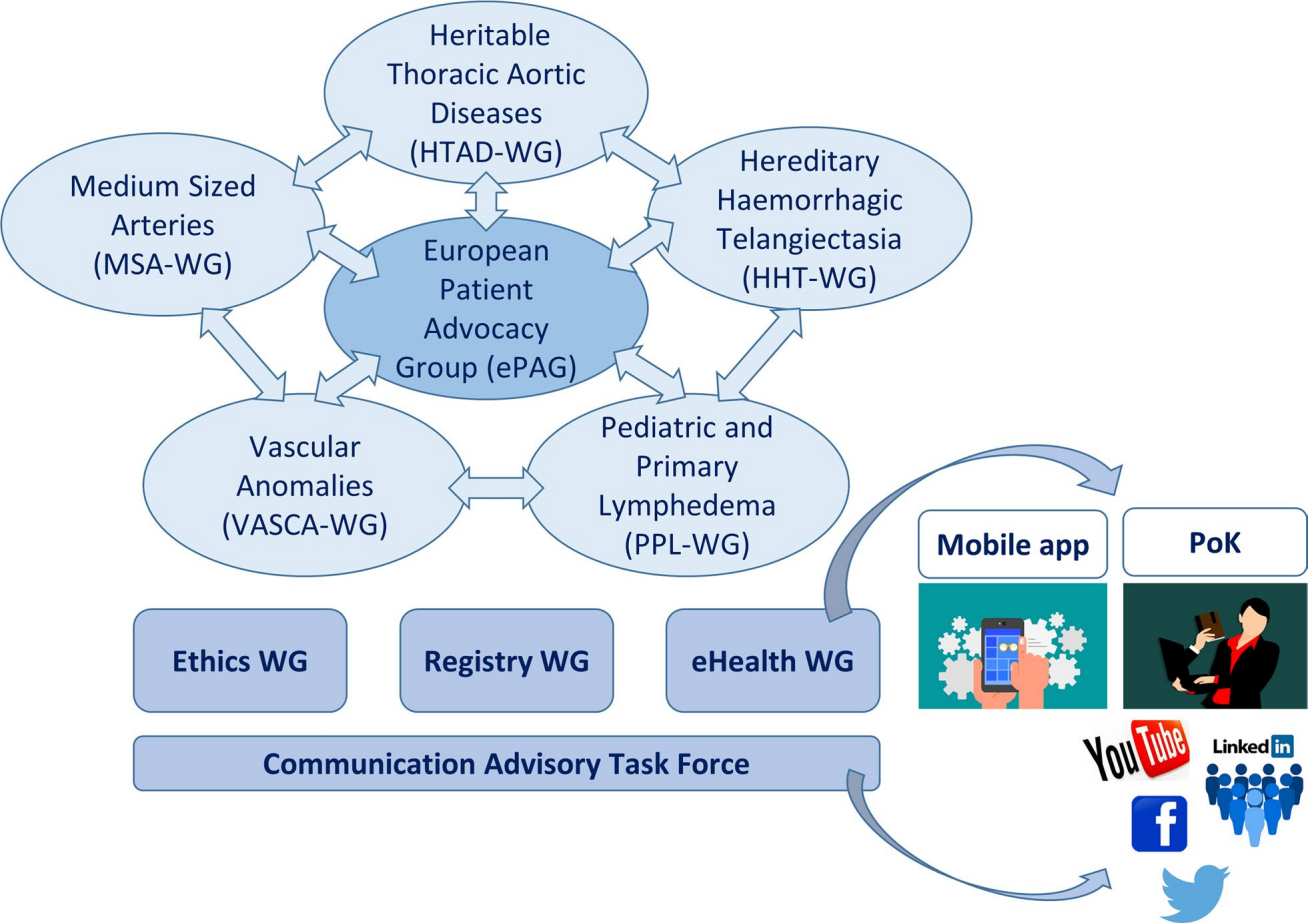
VASCERN structure in the first two years of the ERN lifetime. VASCERN is organized into: five interconnected RDWGs (Rare Diseases Working Groups), the European Patient Advocacy Group (ePAG) at the centre of the ERN, and three transversal WGs: ethics, registry, and eHealth and the Communication Advisory Task Force, managed by the VASCERN coordination team. The figure also summarizes the main actions of the eHealth WG in collaboration with the coordination team and the ERN as a whole: development of a mobile app and development of pills of knowledge (PoK) delivered via digital media

The ERN on Rare Multisystemic Vascular Diseases (VASCERN) addresses complex disorders and conditions that affect different types of vessels and are associated with multisystemic consequences [18, 19]. VASCERN is a multidisciplinary network of HCPs and Patient Organizations (POs) that gathers 35 highly specialized multidisciplinary expert centres from 30 HCPs from 11 countries (Belgium, Denmark, Finland, France, Germany, Hungary, Ireland, Italy, The Netherlands, Sweden, and the United Kingdom) and more than 70 POs from 16 countries. The structure of VASCERN is summarized in Fig. 1. The ERN is organised into thematic Working Groups (WGs):

- five RDWGs (Rare Diseases Working Groups), dedicated to patient care in five different RD areas, with the aim of giving advice on challenging patient cases, collaborating on research, sharing expertise, and writing recommendations [18–23];
- the European Patient Advocacy Group, gathering patient representatives and POs from across the different RD areas to work together on issues of common interest, to maximize the interaction between VASCERN and European POs, to ensure a patient-centred approach, and to disseminate information about health policies, good practices, and recommendations directly to the patients and their families;
- three transversal WGs dedicated to general crossover activities: ethics, registry, and eHealth. The eHealth WG was established with the aim to develop guiding principles, technical specifications, requirements, as well as strategies for effective use of eHealth tools in the context of the ERN; and
- the Communication Advisory Task Force, managed by the VASCERN coordination team.

## The experience of the eHealth WG of VASCERN

The eHealth WG was devised as a central element in VASCERN to ensure effective use of digital technology within the ERN, and, in a broader context, to address the potential of eHealth for the benefit of stakeholders, the public, health policy makers, researchers and HCPs in the area of RDs, as well as the other ERNs [24]. Effective use of digital technology is crucial to enhance the intrinsic value of the ERNs and the expected benefits in the areas of service delivery, patient pathways, scientific evidence updates, patient support, training and learning [25]. The WG adopted a broad definition of eHealth in line with the World Health Organization (WHO) definitions and in line with the 2016 position statement of the European Society of Cardiology [26]. Specifically, eHealth is defined within the WG as the use of information and communication technology—locally and at a distance—to deliver information, resources and services related to health which may reach a wide population, in a personalized manner [27, 28].

### Guiding principles for effective use of eHealth: collaboration and patient-centredness

The eHealth WG of VASCERN was created to bridge the overarching framework of the European policies on eHealth to the actual needs of patients and HCPs, in a *collaborative* and *patient-centred* way. Specifically, the aim of the WG was to identify and implement practical actions to support patient awareness and cross-border healthcare.

**Fig. 2:**
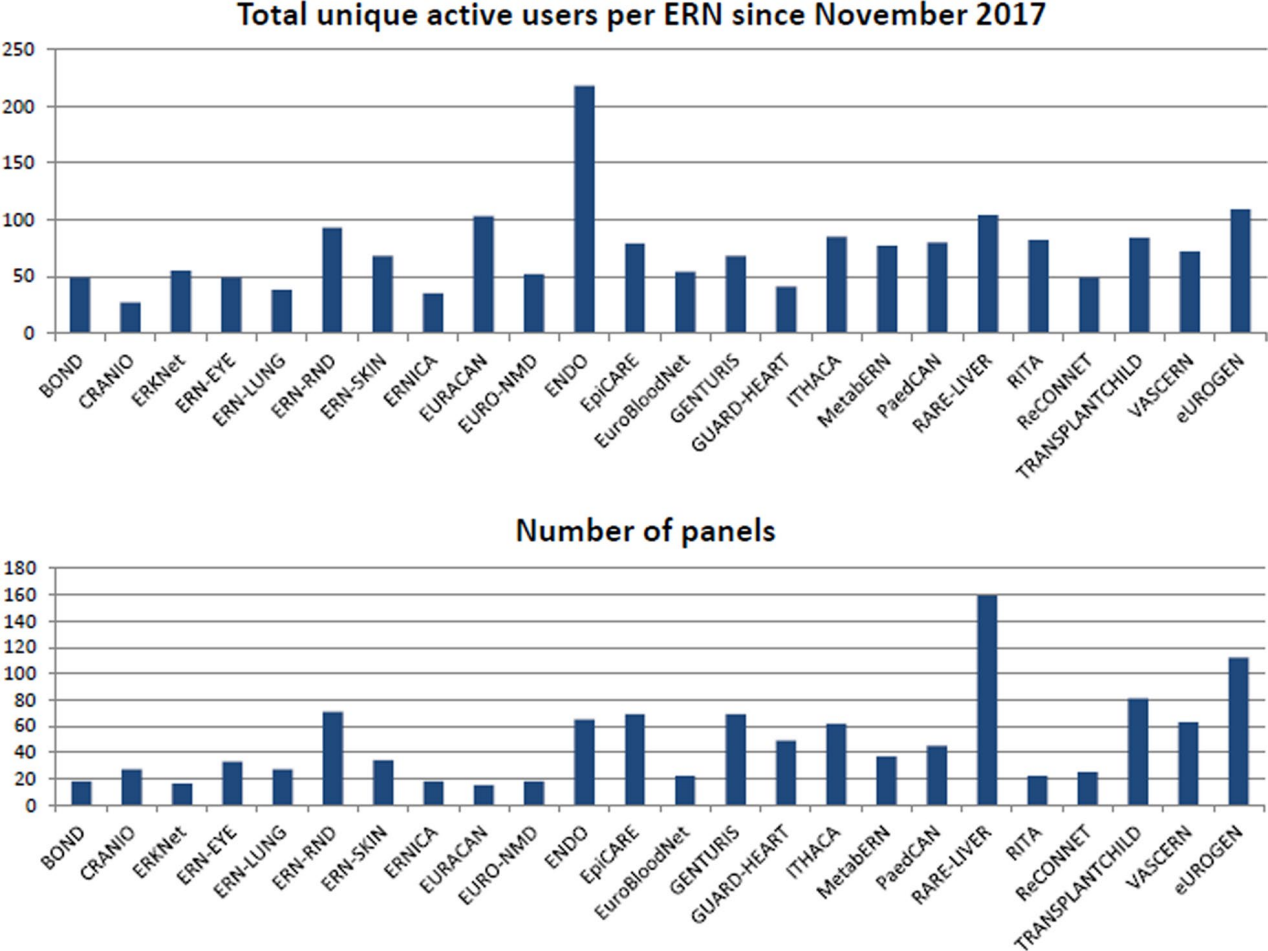
ERNs activity on the CPMS. Top panel: number of unique active users. Bottom panel: number of submitted panels ( source: ERN Project Status Report. June 30, 2020)

A collaborative approach is key to effective eHealth use as, whatever the health policy dimension (local, national, or trans-national) the use of eHealth as a strategic asset demands a coordinated approach. For example, to enable active collaboration between HCPs in the ERNs the EU introduced the Clinical Patient Management System (CPMS). The CPMS is a cross-border web-based platform where HCPs from across Europe can share and discuss challenging cases and share expertise with the aim of advancing knowledge on rare or low prevalence complex diseases for the benefit of patients. In VASCERN, there are currently 80 registered users on the CPMS across the 35 participating expert centres. To date, within VASCERN 49 challenging cases have been discussed and solved on the CPMS across the five RDWGs (25 cases related to paediatric and primary lymphedema, 10 to medium sized arteries abnormalities, 10 to heritable thoracic aortic diseases, and 4 related to vascular anomalies and hereditary haemorrhagic telangiectasia). An analysis of ERNs activity on the CPMS from the official ERN Project Status Report is reported in Fig. 2 (as of June 30, 2020). Figure 2 shows that the number of users and the number of panels vary substantially across the ERNs as the different networks involve a varying number of HCPs. VASCERN is among the top six ERNs in terms of activity on the CPMS in terms of ratio between the number of panels and number of unique active users. Overall, since the launch of the platform in November 2017, there has been a regular increase in interest within VASCERN, and RDWGs that were initially slower in adopting this tool have recently increased their activity on the platform. Therefore, the overall adoption of the CPMS platform is growing and this will translate into increased knowledge sharing and increased number of patient cases successfully addressed through collaboration among experts in the ERN.

Patient-centredness is another pillar of VASCERN and a key principle followed by the eHealth WG. The WG was committed to making the best use of the available tools to answer the specific needs of patients with RD. As reported in the literature, the main patients’ needs can be summarized across five dimensions: coordination of care (e.g., finding an expert centre); diagnostic resources (e.g., getting accurate diagnosis, relying on top level professional education); treatments (e.g., receiving high quality healthcare and prompt emergency care); patient support (e.g., networking with POs, receiving reliable and understandable information); and innovative research (e.g., contributing to innovation) [29–32]. To identify the key actions of the eHealth WG, to be developed during the ERN lifetime, the above general needs were taken into consideration and, specifically, themes related to coordination of care, patient support, and diagnostic resources through professional training and education. This is also in line with the outcomes of a patient focus group organized by the ERN on Intellectual disability, TeleHealth and Congenital Anomalies (ITHACA). The focus group identified key themes in the area of patients’ *access* to experts for diagnosis and guidance to specialists for appropriate care and management, *mapping* relevant services in different countries, and *patient centred* provision of training and education programs for patients, families, and professionals, fully in line with the listed priorities of the eHealth WG [33].

In addition to the above list of needs and themes, a general principle followed by the WG was to facilitate the adoption of the proposed tools by making the information/knowledge available, easy to understand, and usable. Following the above principles, and following a preliminary analysis of potential impact and feasibility within the ERN lifetime, the main objectives set by the WG were defined as:

1. To develop a mobile app to help patients find expert centres and POs (the VASCERN mobile app);
2. To develop resources (Pills of Knowledge, PoK) for training and education via digital platforms (eLearning).

## The VASCERN mobile app

The VASCERN mobile app was devised as a modular platform, to be developed throughout the ERN’s lifetime via incremental upgrades and gradual inclusion of new services. The basic module of the app was designed to include service finder functionalities (finding the closest HCP or PO for a given RD) and it was built following a pilot experience by members of the eHealth WG, i.e. a mobile app (Explo-Rare) launched in 2015 to support patients with RDs to identify clinical centres in the Lombardy Region in Italy [18, 34]. Additional modules of the app will be developed in the future including, for example, personalized digital patient passports, tailored educational material, management of emergency calls, and remote consultation.

The VASCERN mobile app was launched in January 2019 and is available free of charge on the two leading markets, the iTunes app store and the Google Play store. The first version of the app has been developed in English and in August 2020 a second version, including multiple EU languages, has been launched. The app includes a repository and service finder functionality to locate the closest HCPs and POs for a given RD in a given geographical area [34]. Furthermore, to help patients find detailed information about their conditions the app reports the orphacode and a link to the corresponding Orphanet database disease entry page for each RD. In its first version, the app’s repository maps all the HCPs that participate in VASCERN and several POs throughout Europe, both from VASCERN and from outside the ERN. The first version of the app included 35 expert centres and 46 POs from across 16 European countries. The second version includes 76 clinical centres overall, comprising new VASCERN affiliated partner centres and several referral centres that cooperate with VASCERN expert centres, and 67 POs overall.

**Fig. 3:**
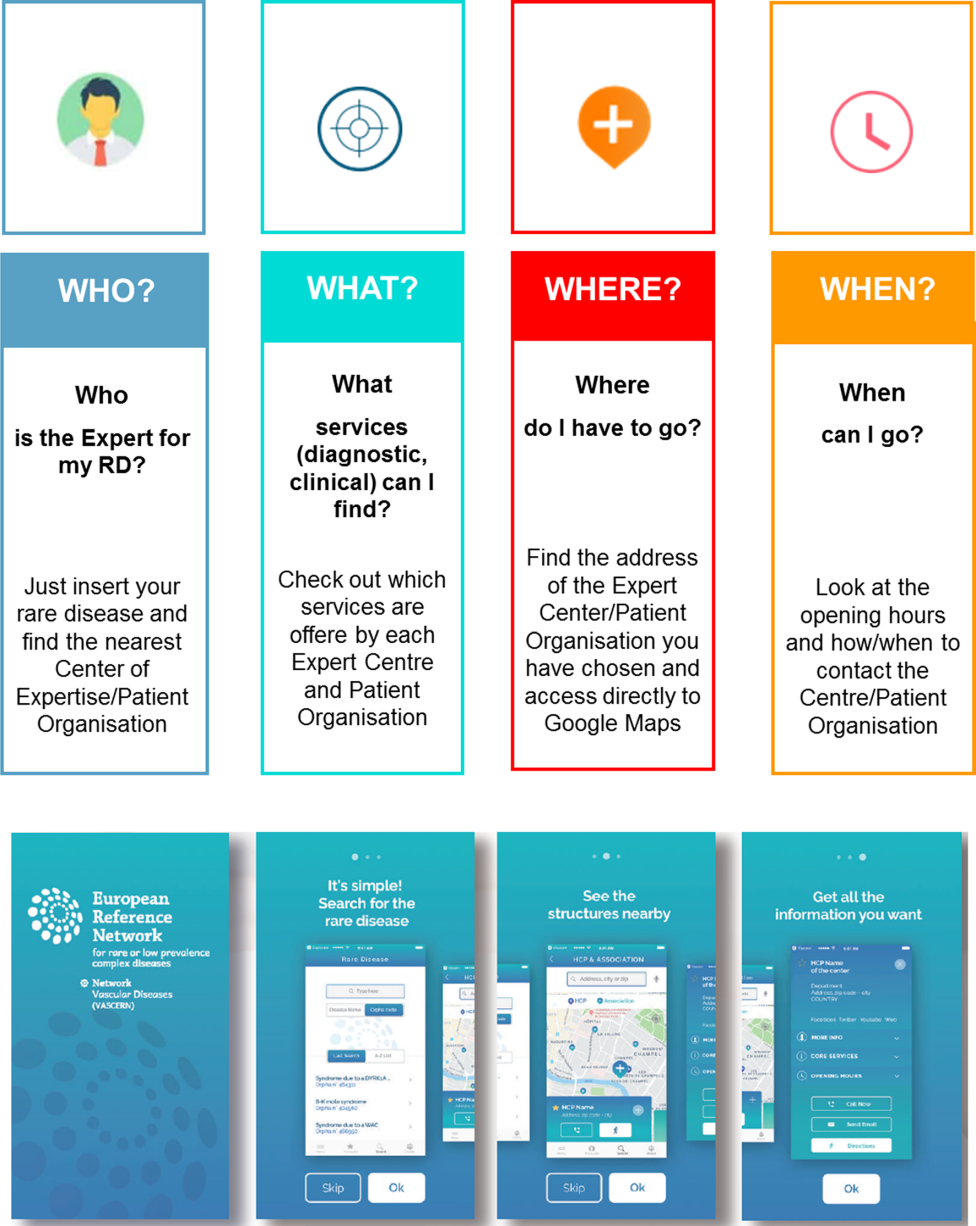
The VASCERN app. Top panel: outline of the main app functionalities. Bottom panel: the app user tutorial, showing a typical usage flow

The app provides simple and essential information. For HCPs, the dataset includes contact details (name, address, contacts, opening hours, coordinator details and specialization), information about the RDs managed by the centre, a list of medical specialties available and services offered (e.g., exams, highly specialized diagnostics, treatments, counselling, emergency call centre), and additional resources available (e.g., websites, social media pages and groups). For POs, the dataset includes contact details (name, address, contacts, opening hours, contact persons), the list of services offered (e.g., help line, connection to social services, training and education), and links to other related POs. The app also shows cross-links between HCPs and POs that have well established collaborations. The app integrates seamlessly with built-in smartphone services and app callouts to provide related functionalities, for example geolocation, phone calls, messaging, or email. Figure 3 shows an outline of the app functionalities and a typical flow, as shown in the app tutorial: selection of the RD of interest, localization of the HCPs and POs in the area of interest, and retrieval of the related information, indications, and contact details.

A direct advantage of this type of app is that, with a simple and easy to use tool, a patient with multisystemic vascular RD can find expert centres and POs in different geographical regions across Europe. Patients can look for clinical centres and POs located not only close to where they live but also, for example, in different countries and regions. This can be useful, for example, when moving to a new place or while traveling. Knowing in advance where to go to receive specialised healthcare can help improve patients’ self-confidence, safety, and quality of care as all the HCPs included in the app have gone through rigorous validation at national level as part of the ERN application process. Furthermore, by gathering information about several POs, including the smaller ones, the app facilitates contact between patients and POs that may otherwise be difficult to find and get in contact to, particularly when the patient is far from home or when the PO does not have a strong present on the web or social media.

The first version of the mobile app was promoted via the ERN website and social media for an initial rollout and testing phase and it has been downloaded by more than 200 patients overall. Following positive feedback, the second version of the app, launched in August 6 2020, will be promoted broadly through the ERN website, social media, POs, expert centres and their national networks. Examples of feedback collected during the rollout and testing phase from patients and clinicians are shown in Table 1.

**Table 1:** Users’ feedback about the VASCERN app

App attributes	Users’ feedback
Value	“The VASCERN application is of great value for patients with rare diseases and for their physicians” “All in all, the VASCERN application fulfils its aims, it can be a great help for patients and physicians”
Ease of use	“The nearest centre with a profile of the particular disorder and its contact information can be found easily. Accessing the required information is straightforward” “The app looks OK, easy and simple to use” “Seems to be user-friendly and well-done” “Good feeling, intuitive”
App functioning	“The offered features like route planning to the Healthcare Provider and directing users to Orphanet for more information on the disease work perfectly well” “Application works without greater problems” “In general very clear and intuitive”

### Future developments

In addition to the service finder and disease information functionalities currently available, further modules will be included in the app. For example, an individualized module with the personal digital patient passport. Conventional patient passports (also referred to as healthcare passports) are small paper documents (wallet-sized or pocket-sized) that people with a RD carry, to be shown to medical staff and doctors whenever needed, especially in case of emergency. Patient passports detail all the necessary information about the patient’s condition, healthcare needs, possible daily life and support mechanisms, and information about who to contact in case of need. Paper-based patient passports have been successfully developed and used in a range of settings by patients with various conditions (e.g., dementia, congenital heart disease, life-limiting diseases, or paediatric conditions), demonstrating a potential to improve quality of care and patient-doctor communication [35–38]. In the area of RDs and vascular RDs, some POs have successfully developed paper-based patient passports, for example the French Association of Ehlers-Danlos Syndromes (AFSED), the German Ehlers-Danlos Initiative, the Loeys-Dietz Syndrome Foundation (LDSF), and the Northern Ireland Rare Disease Partnership (NIRDP). Compared to conventional paper-based versions, digital patient passports can have several advantages. For example, they can be accessed at any time and place through internet connection or stored locally on the phone. Also, they do not tend to deteriorate in time, and are difficult to lose if stored in the smartphone or in the cloud. In addition, digital patient passports allow for the inclusion of a large amount of information compared to paper-based ones, which present limited information to fit the paper size. Moreover, digital patient passports can be personalised and tailored to the patient’s needs, profile, and context, and can be adapted easily over time if needed, in a patient-centred way.

A pilot collaborative study of the eHealth WG and the Medium Sized Arteries WG of VASCERN has shown the feasibility of developing a digital passport or patients with vascular Ehlers-Danlos Syndrome (vEDS) [39]. However, further research is needed as digital patient passports may also pose new challenges, for example in terms of data management, patient privacy, informed consent, and control of shared data. In addition, digital passports may not necessarily fit the needs of every patient with the disease as, for example, people with limited digital skills and people not willing to use a smartphone to handle clinical issues might still prefer to use conventional paper documents. Issues about confidentiality and ethical rules in various EU countries, remain to be solved. Further research and a survey among patients and POs will be needed to collect preferences, suggestions, and minimum requirements, in order to develop digital passports that can be tailored to the needs of the different RDs covered by the ERN.

## Pills of knowledge (PoK) and eLearning

Following the principles of collaboration and patient-centredness, the eHealth WG and VASCERN as a whole invested in the development of educational materials for patients, families, caregivers, and healthcare professionals (Pills of Knowledge, PoK) delivered via digital media (eLearning) and that may can be incorporated as educational utilities in future versions of the mobile app. In fact, due to scarcity of resources, scattered clinical cases, and limited knowledge sources in the area of RD, it is important that healthcare professionals, patients, and families are provided with widely accessible, consistent, easy to understand, and trustworthy information. To answer this need, some POs, HCPs, and medical publishers have developed resources about RDs that include information on RD pathopysiology, diagnosis, clinical management, treatment, as well as advice for daily life and leisure activities [40–43].

In VASCERN, the term PoK encompasses a range of educational materials for various target groups to be delivered via digital media (eLearning) in a collaborative way within the ERN. The role of the RDWGs is to provide medical knowledge, endorse and verify the content of the PoK, and fit the content to different target groups, collaborating with external experts whenever needed. The role of the European Patient Advocacy Group is to provide guidance about PoK directed to patients, families, and caregivers, suggesting solutions based on the target users’ needs. The ERN coordination team manages the validation and publication of PoK, followed by translation, subtitling, and communication-related activities. The role of the eHealth WG was to ensure that PoK are easy to use, easy to understand, and that the format is tailored to the target group and viable for delivery through digital platforms (e.g., websites, social media, webcasting, portals, or mobile apps) so that they can be accessed (and shared) virtually anytime and anywhere.

A large variety of PoK have been planned to be developed throughout the ERN lifetime. Regarding content, the topics identified include, for example, “What is Heritable Thoracic Aortic Disease (HTAD)?”, “What is aortic root replacement?”, “what is the role of genetic counselling and family screening?” and so on. For topics that are relevant to more than one target group, different PoK are planned in order to adapt the content the average level of knowledge of the different groups. Similarly, considering the multisystemic nature of RDs in VASCERN, a range of healthcare professionals can be potentially interested and therefore the PoK content takes into account the different medical specialties. Regarding the formats, PoK are planned in the form of brochures, booklets, short videos (2–5 min), and comics for children. In addition to these ad hoc developed PoK, relevant material from scientific meetings organized by ERN members, lectures, and material provided by POs is used and adapted to the different channels of delivery.

To maximise the potential reach of these materials in different types of audience, several means of delivery are used. PoK are made available on relevant websites (e.g., on the website of VASCERN, the websites of the HCPs and POs participating in the ERN, additional collaborating HCPs and POs in the area of multisystemic vascular RDs, and medical/professional associations), via social media and via professional social networks. The following social media are used by VASCERN to disseminate the PoK to a range of target audiences: Twitter (866 followers), Facebook (741 followers), LinkedIn (256 followers), and YouTube™ (437 followers). These figures demonstrate very good social media presence of the ERN as, for example, considering the number of followers VASCERN is the second top ERN on LinkedIn (the top one, EpiCARE, has 285 followers) and the fourth top ERN on Twitter following EpiCARE (1518 followers), ERN Reconnect (1193 followers), and ERN-RND (1096 followers).

**Fig. 4:**
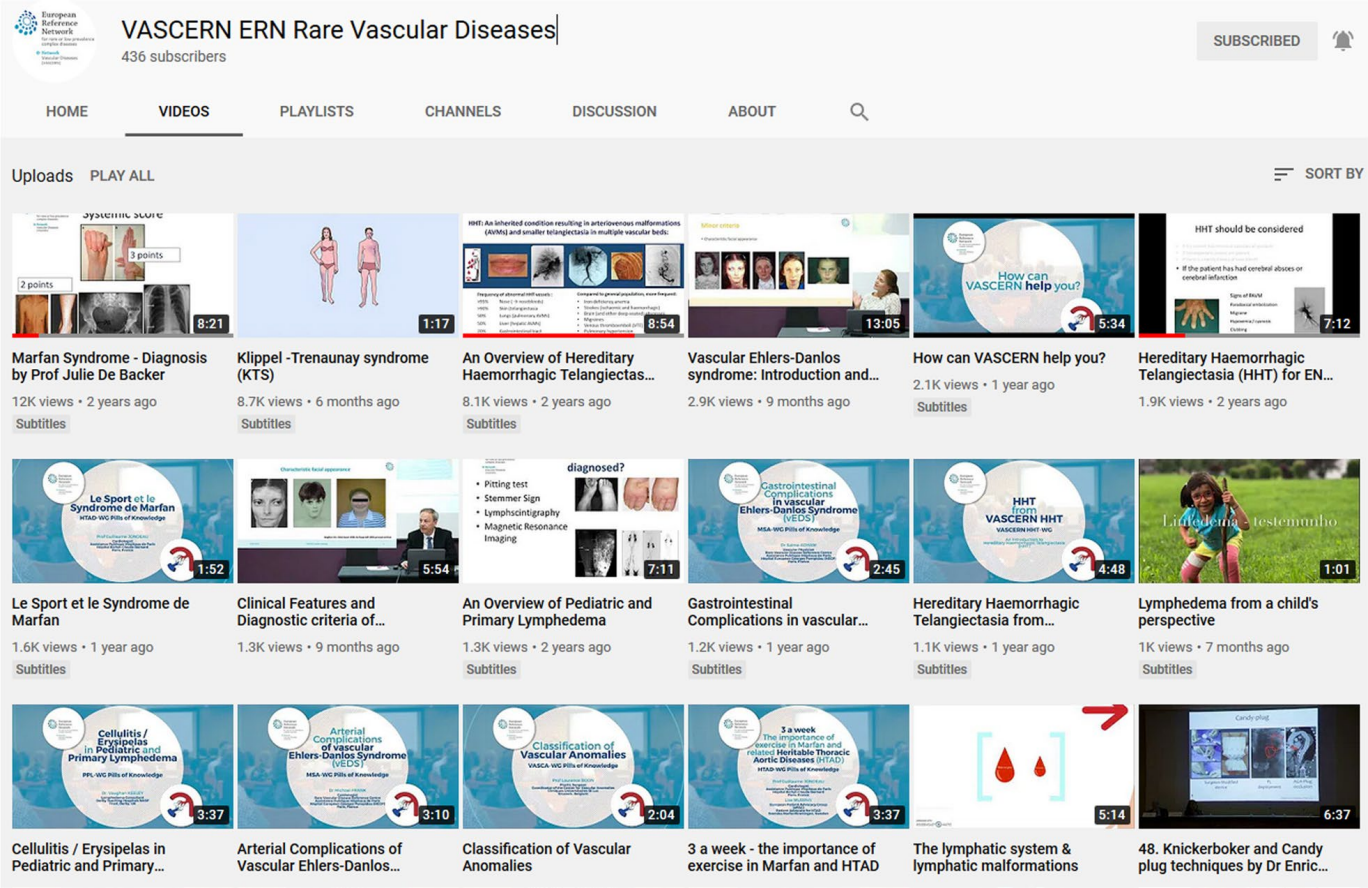
The VASCERN YouTube™ channel

Figure 4 shows the VASCERN YouTube™ channel. The channel contains, to date, more than 100 videos, including various short educational videos as well as longer videos with more detailed content for different target audiences. Since its launch on October 30 2017, the VASCERN YouTube™ channel has had a total of about 60′000 views overall. The average number of views per video is equal to 522 (s.d. = 1625) and the average number of views per video per year is equal to 1650 (s.d. = 12,519), with 12 videos that had more than 1000 views per year. The five most viewed videos are: “Marfan Syndrome—Diagnosis” (about 12,200 views in 2 years and 3 months), “Klippel-Trenaunay syndrome (KTS)” (about 8700 views in one month), “An Overview of Hereditary Haemorrhagic Telangiectasia” (about 8200 views in 2 years and 5 months), “Vascular Ehlers-Danlos syndrome: Introduction and new criteria” (about 2900 views in 8 months), and “How can VASCERN help you?” (about 2100 views in 1 year and 6 months). The topics of the top five videos demonstrate interest in general aspects of specific diseases (pathophysiology and diagnostic criteria) as well as interest into the ERN as a whole.

## Limitations and future work

It is acknowledged that the above described actions carried on by the eHealth WG represent a pilot experience. However, the experience of the eHealth WG provides a picture, albeit preliminary, of the practical steps that can be taken to develop patient-oriented, easy to use, low cost tools. In fact, shortage of funding is a common limitation in the clinic and in research therefore it is important to develop tools that are feasible both in terms of implementation and in terms of delivery. The simplicity and the relatively low cost of the solutions here proposed are the basis for their potential scalability for widespread use. The use of widely available channels for dissemination, such as the web and social media, can partially help fill the gap associated with limited funding. In addition, a lively environment within the ERN, made up by clinical experts who actively collaborate towards a common goal and by typically small, local POs that are eager to collaborate on a cross-national level and work to put the patient at the centre, is essential to make profit of each one’s peculiar expertise and ideas and to overcome limitations due to limited time and limited funding. In this respect, it is essential that the coordinating team and transversal working groups put their efforts into ensuring engagement of the participating institutions, both HCPs and POs, to have them acting as ambassadors in their local networks and communities. Our WG has followed a human-in-the-loop approach that starts from the definition of the individual needs of patients and returns to patients with simple, usable, and patient-centred solutions.

Future work is needed to take full advantage of the potential of eHealth for vascular RDs and, more generally, for patients with RDs. Within VASCERN, it will be important to assess the impact of the mobile app and PoK/eLearning resources more deeply and it will be important to collect specific feedback from users through surveys that can be used to implement strategies to improve these tools based on the users’ needs and expectations and maximize the benefits of these actions. For the VASCERN mobile app, in addition to developing individual digital patient passports and providing personalized access to PoK it will be important to assess the actual reach of the app and to monitor its trends over time, particularly after the launch of the second version as the one here presented represents a pilot experience. It will be important to analyse, once that a significant number of app users will be reached, what is their geographical distribution and what are the practical benefits experienced, i.e. in terms of access to care, acquired knowledge, or self-efficacy. In this context, it will be important to address if, and how, the geographical distribution and the number of patients who use the app and get benefit from it will change following inclusion of additional HCPs and POs in the ERN, for example following the launch of the second version of the app. Gradual inclusion of additional expert centres that are not members of VASCERN but are indeed recognized as part of an official national network when available (e.g., in France) and, in the absence of an official national network, expert centres formally recognized by their peers is foreseen. This will allow to significantly expand the range of clinical services that patients can access to throughout Europe, on the basis of the principle of cross-border care.

In the area of eLearning, further developments of the PoK portfolio will be needed to cover a broader range of topics, formats, target audiences, and languages. Monitoring the impact of these new PoK (number of views, geographical distribution of followers, number of post shares on social media) will be essential to understand the optimal communication strategy and prioritize the PoK content. It will be also important to perform a survey among patients and healthcare professionals to address the learning outcomes of the PoK delivered and to understand how healthcare professionals can incorporate these eLearning tools into their daily clinical practice.

Overall, the work carried out by the eHealth WG of VASCERN represents a pilot experience that has showed how patient-centred eHealth solutions can be practically developed in a collaborative manner and made widely available for the benefit of patients. This experience may serve as a model for other ERNs and, more generally, for the RD international community as the tools described in this article may be adapted to the needs of different RDs. Further research is needed to monitor the actual patterns of patients’ access to care through ERNs that may be supported by tools such as the app here developed. Moreover, a Europe-wide effort is needed to measure the quality of health information and healthcare services that patients receive since the launch of the ERNs in Europe. Assessment of benefits should consider not only the medical and clinical outcomes domains but, also, overall patient wellbeing and satisfaction quality of life as well as awareness, self-confidence, efficacy, and engagement to understand how these outcomes indicators may change when accessible, easy to use, easy to understand digital tools are made available directly to the patients.

## Conclusions

- Cross-border cooperation in the area of RDs, as promoted by the recent EU policies, has led to the establishment of ERNs in different RD areas. ERNs are designed as newly available resources for patients with complex, unmet needs as they can partially overcome some of the obstacles that RD patients face due to the geographical dispersion of expert centres. By pooling knowledge and expertise across the EU, ERNs may contribute to easier access to diagnosis, quicker treatment and higher quality of healthcare throughout the EU. However, in order to realize the full advantage of this potential, further efforts will be needed in the medium and long term through specific research funding, industry involvement, and professional education
- Within VASCERN, the eHealth WG has been set up to advance the use of eHealth and mHealth to develop, in a collaborative way, novel patient-centred tools capable of providing information, training, and services to RD patients and healthcare professionals.
- The eHealth WG has developed the first module of a mobile app that can be used to locate HCPs and POs for various RDs across Europe and to get general information about multisystemic vascular RDs. By providing patients with up-to-date information about where and how to find what they need, the app will contribute to improve healthcare for patients with RDs, in line with recent EU policies. Preliminary data collected in the app rollout and testing phase demonstrated the feasibility and value of the approach. Moreover, thanks to its modular nature the app is open to further developments (e.g., digital patient passports, inclusion of eLearning features).
- The eHealth WG has contributed to the development of several Pills of Knowledge (PoK) for various target groups and in various formats delivered via a range of digital platforms. Preliminary results showed that the PoK can potentially reach a large audience and that there is substantial interest in the areas of RD pathophysiology and diagnosis.
- The eHealth WG experience within VASCERN has shown that successful use of eHealth in the area of RDs requires more than just the development of new tools and that a collaborative approach is key to success. Additional key issues, such as implementation strategies, heterogeneity of systems across countries, legal and ethical concerns, and data protection issues, still need to be addressed to improve coordination and information sharing between HCPs, POs, and policy makers.
- The pilot experience of the eHealth WG can serve as a reference model and can potentially be scaled up to leverage the impact of the VASCERN and, more generally, of ERN and international collaborative networks in terms of improved access to diagnosis, treatment, and high-quality healthcare for patients with multisystemic vascular RDs.

## Data Availability

Not applicable.

## Abbreviations

ERN: European Reference Network
EU: European Union
HCP: Healthcare Provider
PO: Patient Organization
PoK: Pills of knowledge
RD: Rare disease
VASCERN: European Reference Network on Rare Multisystemic Vascular Diseases
WG: Working group
